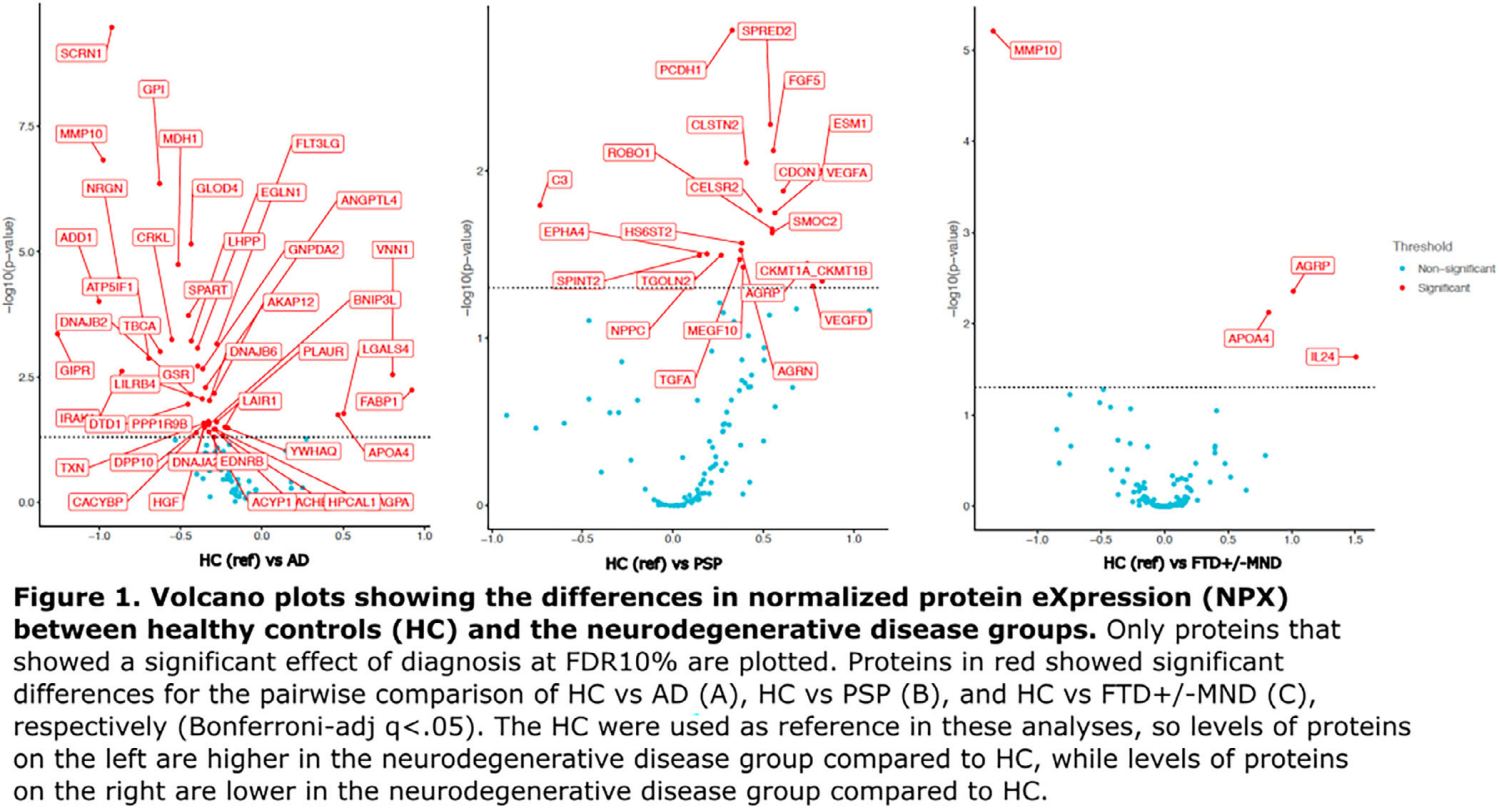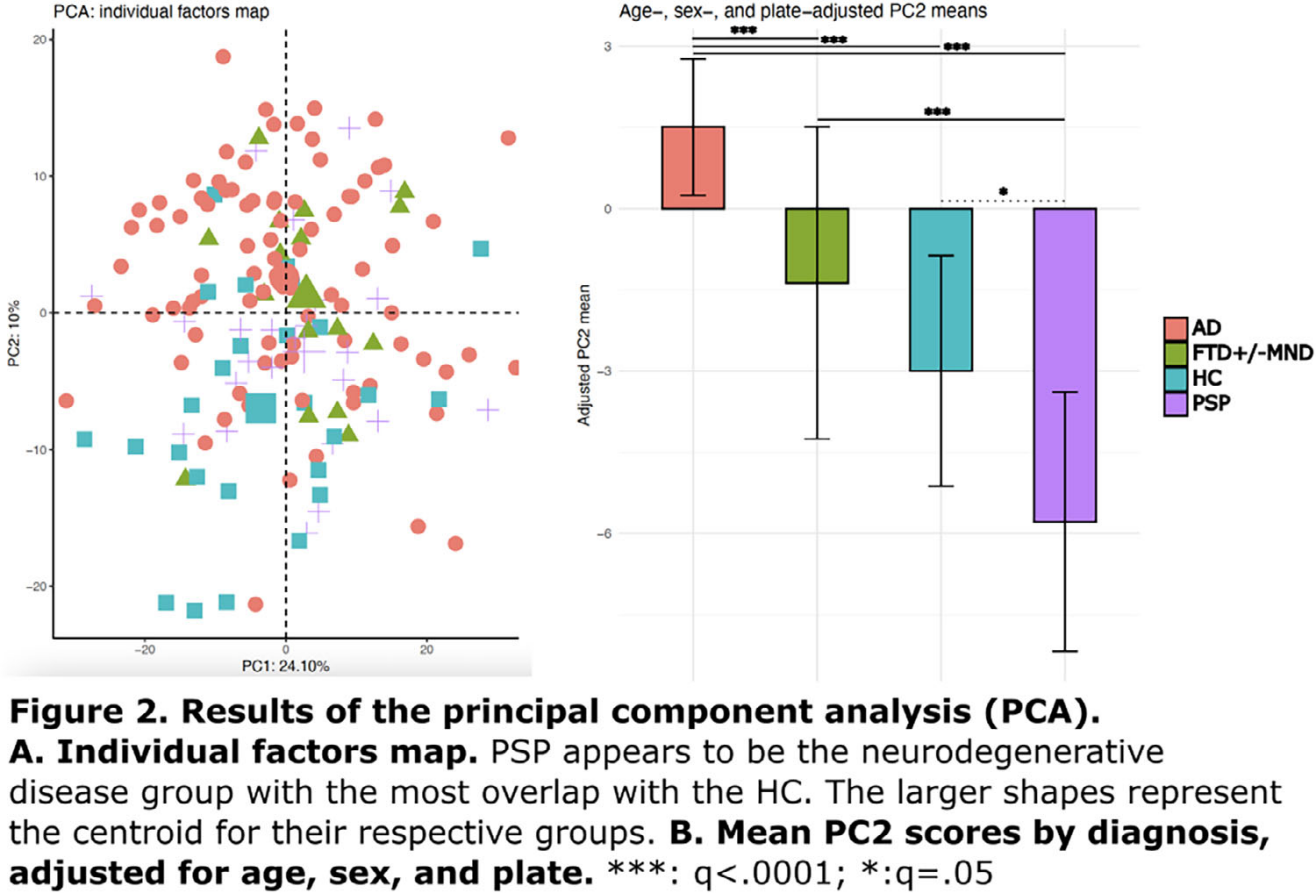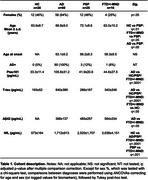# Characterizing the relationship between neuroinflammation and neurodegeneration in AD and FTLD

**DOI:** 10.1002/alz70856_107533

**Published:** 2026-01-09

**Authors:** Chloe Anastassiadis, Simrika Thapa, Anna Vasilevskaya, Kasey Cortez, Nico Paulo Dimal, Michelle Tsang, Blas Couto, David F. Tang‐Wai, Susan Fox, Gabor G. Kovacs, Anthony E. Lang, Pia Kivisäkk, Bradley T. Hyman, Steven E Arnold, Martin Ingelsson, Carmela Tartaglia

**Affiliations:** ^1^ Memory Clinic University Health Network, Toronto, ON, Canada; ^2^ Tanz Centre for Research in Neurodegenerative Diseases, University of Toronto, Toronto, ON, Canada; ^3^ Institute of Medical Science, University of Toronto, Toronto, ON, Canada; ^4^ Krembil Brain Institute, University Health Network (UHN), Toronto, ON, Canada; ^5^ Rossy PSP Program, University Health Network, Toronto, ON, Canada; ^6^ University Health Network, Toronto, ON, Canada; ^7^ Memory Clinic, Toronto Western Hospital, University Health Network, Toronto, ON, Canada; ^8^ UHN Memory Clinic, Toronto, ON, Canada; ^9^ Instituto de Neurología Cognitiva (INECO), CABA, Buenos Aires, Argentina; ^10^ The Edmond J. Safra Program in Parkinson's Disease and Morton and Gloria Shulman Movement Disorders Clinic, Toronto, Toronto, ON, Canada; ^11^ The Edmond J. Safra Program in Parkinson's Disease and Morton and Gloria Shulman Movement Disorders Clinic, Toronto, ON, Canada; ^12^ Department of Laboratory Medicine and Pathobiology, University of Toronto, Toronto, ON, Canada; ^13^ Rossy PSP Program, University Health Network and the University of Toronto, Toronto, ON, Canada; ^14^ Krembil Brain Institute, Toronto, ON, Canada; ^15^ Movement Disorder Clinic, Toronto Western Hospital, University Health Network, Toronto, ON, Canada; ^16^ Harvard Medical School, Boston, MA, USA; ^17^ Massachusetts General Hospital, Harvard Medical School, Boston, MA, USA; ^18^ Massachusetts General Hospital, Boston, MA, USA; ^19^ Tanz Centre for Research on Neurodegenerative Diseases, Toronto, ON, Canada; ^20^ Department of Public Health and Caring Sciences, Uppsala University, Sweden, Sweden; ^21^ Krembil Brain Institute, UHN, Toronto, Toronto, ON, Canada; ^22^ University of Toronto, Toronto, ON, Canada; ^23^ Rossy Progressive Supranuclear Palsy Centre, University Health Network and the University of Toronto, Toronto, Toronto, ON, Canada

## Abstract

**Background:**

Although immune dysregulation has been reported in many neurodegenerative diseases (NDDs), our understanding of shared vs disease‐specific features is still lacking. Here, we analyze a large panel of inflammatory markers in Alzheimer's disease (AD) and frontotemporal lobar degeneration (FTLD)‐related syndromes.

**Method:**

The cohort included 26 healthy controls (HC); 90 biomarker‐positive AD patients (including 57 young‐onset); 25 progressive supranuclear palsy (PSP) patients; and 16 patients clinically diagnosed with semantic variant primary progressive aphasia or frontotemporal dementia with motor neuron disease (FTD+/‐MND group). Their CSF samples were tested for inflammation (737 proteins, Olink proximity extension assay) and neurodegeneration biomarkers (NfL, Aβ42, ptau181, total tau). All analyses were corrected for age, sex, and plate.

**Result:**

ANCOVAs showed alterations in distinct subsets of proteins in NDDs compared to HC: the AD group was characterized by increased levels of inflammatory markers, while the opposite was seen in PSP. The smaller FTD+/‐MND cohort only showed differences in four proteins (Figure 1). Gene‐set enrichment analysis (GSEA) highlighted the implication of cell signaling pathways (including the transmembrane receptor protein tyrosine kinase signaling (q<.05) and response to growth factor (q<.10) pathways) in PSP compared to HC.

Principal component analysis (PCA) revealed a limited overlap between NDDs and HC (Figure 2). Differences between diagnoses were best captured by PC2 (10% variance). Among the biomarkers, ptau181 was the strongest correlate of PC1 (24% variance) and PC2 (q<.0001).

In preliminary investigations of astrocytic contributions to these differences, YKL‐40 levels (astrocytic reactivity) were measured for 22 PSP subjects. YKL‐40 and ptau181 were associated with the levels of distinct sets of proteins (after correcting for disease duration and age at onset). In patients with low vs high YKL40, pathways related to white blood cell function (e.g. lymphocyte and neutrophil chemotaxis, chemokine binding) were the top differentially expressed pathways (q<.01).

**Conclusion:**

There are distinct inflammatory patterns in AD, PSP, and FTD+/‐MND. AD is characterized by increases in inflammatory marker levels, while in PSP the opposite is seen. These differences appear to be related to ptau181‐related pathology. Future directions include assessing the contributions of known mediators of neuroinflammation, such as astrocytic reactivity, APOE genotype, and age at onset, to these differences.